# Physical Activity, Empowerment of the Immune System and Public Health: What We Learned from the COVID-19 Pandemic

**DOI:** 10.3390/ijerph192113837

**Published:** 2022-10-24

**Authors:** Francisco José Gondim Pitanga

**Affiliations:** Postgraduate Program in Rehabilitation Sciences, Institute of Health Sciences, Federal University of Bahia (UFBA), Salvador 40110060, Brazil; pitanga@lognet.com.br

The importance of physical activity for the cardiovascular, metabolic and mental health systems with its repercussions for public health has been studied for some time, although further studies are needed due to the depletion of health services observed during the COVID-19 pandemic. On the other hand, little attention has been paid to the importance of physical activity for the empowerment of the immune system and how it can impact health systems around the world.

The empowerment of the immune system, through the practice of physical activity, to minimize the adverse effects of the infectious process caused by the “SARS-CoV-2” virus has been reported since the beginning of the COVID-19 pandemic [1]; however, the first articles on the topic were only recently published, which reported a reduction in aggravation of the disease, reduction in hospital admissions and a decrease of death from COVID-19, as well as a reduction in the possibility of infection by the “SARS-CoV-2” virus [2,3,4,5].

In this way, it was suggested that, the conflict between the virus and the immune cells of our body, during the infectious process caused by the new coronavirus, there would be a release of pro-inflammatory cytokines, which, when directed to the lungs, could cause a greater severity of disease, often requiring more drastic interventions, such as mechanical ventilation [1].

Thus, more physically active individuals could present more adequate defense mechanisms, such as an increase in the immune cells of the innate defense system, in addition to the proliferation of lymphocytes, which could contribute to reducing the inflammatory process caused by the conflict between the virus and the immune cells of our body. Therefore, reducing the inflammatory process in the lungs could reduce the severity of the patient’s clinical condition, avoiding the use of more drastic interventions, with a consequent reduction in the burden on health systems [1].

Furthermore, regarding the fact that physical activity also prevents “SARS-CoV-2” infection, observed in recent publications [4,5], the mechanisms can be partially attributed to the higher concentration of immune cells, such as T lymphocytes, in addition to the increased resistance of the mucosal immune barrier (salivary IgA immunoglobulin), observed in more physically active individuals [6,7,8], since it has the potential to provide greater immunity against different types of viruses and bacteria that enter the human body through the oral cavity and upper airways [9].

In this way, strengthening the immune system, through physical activity, can be an excellent strategy to minimize the deleterious effects of COVID-19, as well as new viral infections that could affect humanity. With the population becoming more physically active, the depletion of health systems observed around the world could probably have been minimized (Figure 1). More aggressive public policies to promote physical activity are suggested to increase the prevalence of physical activity in the world population, so we will be better protected against future infectious processes with characteristics similar to COVID-19, a fact that can probably reduce the burden on health systems around the world.

## Figures and Tables

**Figure 1:**
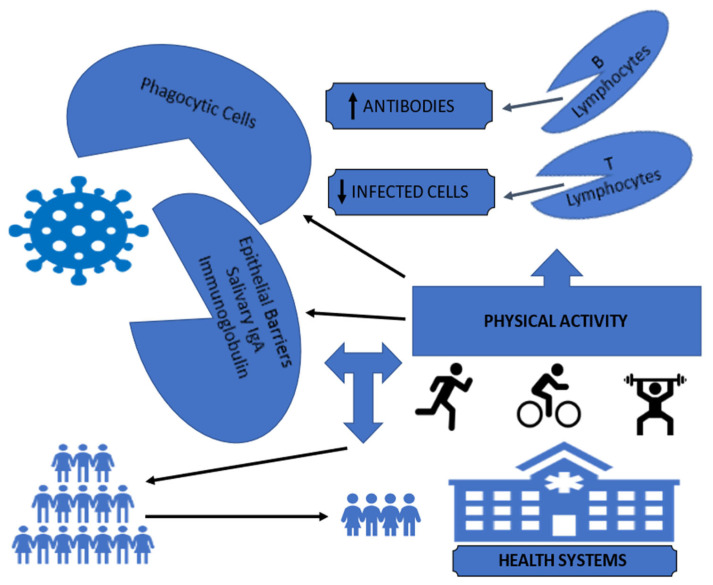
Empowerment the immune system through physical activity with a consequent reduction in the burden on health systems.
